# Current Status and Perspectives of Human Mesenchymal Stem Cell Therapy 2020

**DOI:** 10.1155/2022/9801358

**Published:** 2022-02-11

**Authors:** Jane Ru Choi, Kar Wey Yong, Hui Yin Nam

**Affiliations:** ^1^University of British Columbia, Centre for Blood Research, Life Sciences Centre, 2350 Health Sciences Mall, Vancouver, BC, Canada V6T 1Z3; ^2^Department of Surgery, Faculty of Medicine & Dentistry, University of Alberta, Edmonton, AB, Canada T6G 2R3; ^3^Tissue Engineering Group, Department of Orthopaedic Surgery (NOCERAL), Faculty of Medicine, University of Malaya, Lembah Pantai, 50603 Kuala Lumpur, Malaysia

Mesenchymal stem cell (MSC) therapy shows a great promise for the treatment of injuries and diseases in regenerative medicine [1, 2]. MSCs have the ability to self-renew and differentiate into multiple lineages, making them an ideal candidate for cell therapy [3, 4]. They exist in adult tissues of different sources such as fat, bone marrow, umbilical cord and menstrual blood [5]. In addition to multilineage potential, these cells have the capability of secreting anti-inflammatory molecules and bioactive factors [6]. Therefore, they are commonly used in clinical settings to treat illnesses including autoimmune, inflammatory, degenerative, musculoskeletal and respiratory diseases [7, 8]. While numerous studies reported the clinical applications of MSCs, multiple challenges are yet to be addressed to achieve successful clinical translations. This special issue underlines the most recent advances in therapeutic applications of MSCs. It also discusses many emerging approaches in enhancing the therapeutic effects of MSCs especially in bioprocessing, assessment of efficacy and safety, and clinical delivery strategies.

A total of 16 articles introduced the therapeutic applications of MSCs for diverse types of diseases, including cancers, respiratory, neurological and ocular diseases. A number of comprehensive review articles highlighted the current status and perspectives of MSC therapy in neurological, ocular, and respiratory diseases along with major challenges of translating the research findings into clinical practice. D. Han et al. reviewed the effects of MSC-mediated mitochondrial transfer on inflammatory processes, cell metabolism, survival, proliferation, and differentiation. They also summarized therapeutic potential of MSC-mediated mitochondrial transfer in neurological diseases such as stroke and spinal cord injury. B. Xie et al. performed meta-analysis on randomized controlled clinical trials of cerebral palsy (CP) to evaluate the efficacy and safety of transplantation of human MSCs in children with CP. The therapy increased gross motor function measure scores and comprehensive function assessment up to 12 months with minimal adverse effects. J. S. Nurković et al. reviewed phenotypic characteristics of limbal epithelial stem cells and corneal stromal stem cells and their therapeutic potential in corneal regeneration. Understanding the phenotypic and functional characteristics of corneal stem cells could improve medical and surgical managements of ocular surface disorders. L. Sun et al. reviewed the recent advances of MSCs in allergic rhinitis therapy. Discussing the roles and mechanisms of MSC immunomodulatory effects allows readers to better understand the potential of MSCs in allergic rhinitis therapy.

D. Rady et al. reviewed the major challenges in clinical applications of human MSCs, including donor-related factors, cell source, discrepancies in cell isolation and culture procedures, risk of tumorigenicity, variability in methods of cell delivery, and alteration of MSC properties in response to various drugs and growth factors. By overcoming these challenges, MSC-based therapies could be successfully translated into clinical practices for many unmet medical conditions. M. N. F. B. Hassan et al. systematically reviewed the bioprocessing strategies for large-scale expansion of MSCs. Specifically, the large-scale expansion of 7 different sources of MSCs using 4 different bioprocessing strategies, including bioreactor, spinner flask, roller bottle, and multilayered flask, were comprehensively discussed. It was suggested that the optimization of key parameters, including cell seeding density, oxygen partial pressure and medium formulation is crucial to ensure the development of a sustainable and reproducible platform for utilizing MSCs in clinical settings. These bioprocessing approaches show tremendous potential for large-scale expansion of MSCs without compromising cell quality.

A number of research articles in this issue have revealed the potential therapeutic applications of MSCs for several diseases, including breast cancer, skin cancer, allergic airway inflammation, and Alzheimer's disease. Y. Jiao et al. reported the roles of NLRP1 and CASP4 genes in pyroptosis of breast cancer cell line MCF7 induced by bioactive factors secreted by human umbilical cord-derived MSCs (UCMSCs). It was found that NLRP1 interacts with the adapter protein ASC to form an inflammasome complex, which involves in MCF7 cell pyroptosis. Additionally, neither NLPR1 knockdown nor CASP4 knockdown inhibited the hUCMSC-induced pyroptosis in MCF7, indicating that when one pathway was inhibited, the pyroptosis occurred via another pathway. These findings suggest that elucidating the precise mechanism of hUCMSC-induced pyroptosis in MCF7 could aid in the identification of potential therapeutic agents for breast cancer. D. Miloradovic et al. studied the effects of bone marrow-derived MSCs on anti-melanoma immunity. It was reported that these MSCs play a different role at different stages of melanoma growth. The MSCs showed tumor-suppressive effects at the initial stage of melanoma while opposite effects were shown at the later stage. Therefore, the optimal MSC administration timing is critical for efficient modulation of cancer progression. S. Kim et al. demonstrated the use of extracellular vesicles harvested from adipose-derived stem cells (ASC-derived EVs) to suppress allergic airway inflammation in the mouse models of allergic airway inflammation. It was found that ASC-derived EVs ameliorated allergic airway inflammation through differential expressed genes in the lung such as PON1, Bex2, Igfbp6, Fpr1, and Scgb1c1. In order to study the role of MSC therapy in Alzheimer's disease (AD), H. Lim et al. investigated the inhibition of hyperphosphorylation of tau using human UCMSCs. The authors reported that administration of these cells mitigated the hyperphosphorylation of tau in the AD mouse models through galectin-3 secretion, ameliorating the spatial learning and memory impairments.

Other research articles have improved the knowledge-based of MSCs in several aspects, including characteristics of MSCs isolated from breast cancer patients, efficacy and safety of MSCs in healthy volunteers, proangiogenic activity of MSC secretome, influences of antihypertensive medications on MSCs, development of inducible human MSCs, and effects of mechanical strain on tenogenic differentiation of MSCs. P. Thitilertdecha et al. reported the in-depth characteristics of human ASCs in fresh stromal vascular fraction isolated from breast cancer patients. It was shown that fat tissues collected from the patients contain ASCs with a highly homogenous phenotype similar to the classical bone marrow-derived MSC, which is essential for future investigation of its therapeutic approaches. S. Chin et al. assessed the efficacy and safety of intravenous infusion of allogenic human UCMSCs (CLV-100) in healthy volunteers. Six months after the infusion, subjects infused with a high dose of CLV-100 had higher levels of anti-inflammatory markers (IL1-RA and IL-10) and a lower level of pro-inflammatory marker TNF-*α* compared to those infused with a low dose of CLV-100. Additionally, all subjects did not have any adverse reactions, suggesting that CLV-100 infusion is safe and beneficial for tissue repair and healing. C. M. Chinnici et al. reported that proangiogenic activity of human fetal dermal cell secretome is mainly contributed by EVs. Depletion of EVs from the secretome was found to impair its ability to induce angiogenesis. Additionally, it was shown that more microRNAs with a validated role in angiogenesis were highly expressed in fetal dermal cell-derived EVs compared to adult dermal cell-derived EVs, suggesting that fetal dermal cell-derived EVs are more effective than their adult counterpart in inducing angiogenesis. N. Satani et al. showed that antihypertensive medications such as losartan, captopril, and atenolol with doses prescribed for stroke patients altered immunomodulatory effects of human MSCs. These findings suggest that the effects of antihypertensive drug on MSCs should be taken into consideration for stroke patients receiving MSC therapy. Chen et al. developed inducible human MSC lines to be employed for studies of specific gene activation or inhibition. They found that human MSC-CRISPRi and human MSC-CRISPRa could be useful in studying genes and genetic pathways regulating lineage-specific differentiation of human MSCs. Nam et al. reported that 8% tensile strain at 1 Hz increased expression levels of tenogenic markers and *α*-subunit of the epithelium sodium channel (ENaC) in human MSCs. Expression levels of tenogenic markers in MSCs were decreased when ENaC function was inhibited, suggesting that ENaC plays a key role in mechanical strain-mediated tenogenic differentiation of MSCs.

In short, this special issue has given outstanding insights into the therapeutic effects of MSC on numerous diseases and highlighted the remaining obstacles and potential approaches to translate the research findings into clinical applications. With many opportunities and remaining challenges, we envision that there will be more studies focusing on resolving the challenges to improve the effectiveness of MSC therapy in the near future.

*Jane Ru Choi* *Kar Wey Yong* *Hui Yin Nam*
